# C4.4A as a candidate marker in the diagnosis of colorectal cancer

**DOI:** 10.1038/sj.bjc.6604012

**Published:** 2007-10-02

**Authors:** C Paret, D Hildebrand, J Weitz, A Kopp-Schneider, A Kuhn, A Beer, R Hautmann, M Zöller

**Affiliations:** 1 Department of Tumour Progression and Immune Defence, German Cancer Research Centre, Heidelberg, Germany; 2 Department of Surgery, University of Heidelberg, Heidelberg, Germany; 3 Department of Biostatistics, German Cancer Research Centre, Heidelberg, Germany; 4 Department of Immunogenetics, German Cancer Research Centre, Heidelberg, Germany; 5 Department of Urology, University of Ulm, Ulm, Germany; 6 Department of Applied Genetics, University of Karlsruhe, Karlsruhe, Germany

**Keywords:** C4.4A, galectin-3, colorectal cancer, glycosylation, antigen shedding

## Abstract

C4.4A is a member of the Ly-6 family with restricted expression in non-transformed tissues. C4.4A expression in human cancer has rarely been evaluated. Thus, it became important to explore C4.4A protein expression in human tumour tissue to obtain an estimate on the frequency of expression and the correlation with tumour progression, the study focusing on colorectal cancer. The analysis of C4.4A in human tumour lines by western blot and immunoprecipitation using polyclonal rabbit antibodies that recognize different C4.4A epitopes revealed C4.4A oligomer and heavily glycosylated C4.4A isoform expression that, in some instances, inhibited antibody binding and interaction with the C4.4A ligand galectin-3. In addition, tumour cell lines released C4.4A by vesicle shedding and proteolytic cleavage. C4.4A was expressed in over 80% of primary colorectal cancer and liver metastasis with negligible expression in adjacent colonic mucosa, inflamed colonic tissue and liver. This compares well with EpCAM and CO-029 expression in over 90% of colorectal cancer. C4.4A expression was only observed in about 50% of pancreatic cancer and renal cell carcinoma. By *de novo* expression in colonic cancer tissue, we consider C4.4A as a candidate diagnostic marker in colorectal cancer, which possibly can be detected in body fluids.

C4.4A is a highly glycosylated glycosylphosphatidylinositol (GPI)-anchored protein with 30% homology to the urokinase receptor (uPAR) (Rösel *et al*, 1998). C4.4A, also described as Ly6/PLAUR domain containing 3, and uPAR belong to the Ly-6 family, characterised by repetitive cysteine residues which allow a typical ‘three finger’ folding via disulphide bridges (Tsetlin, 1999). While uPAR has three cysteine containing domains, C4.4A has two and the third domain is devoid of cysteines (Rösel *et al*, 1998). Most other members of the Ly-6 family have only one domain (Ploug and Ellis, 1994).

The C4.4A protein was first identified in a highly metastasizing rat pancreatic adenocarcinoma line (Matzku *et al*, 1989). In the rat, C4.4A (rC4.4A) is strongly upregulated during implantation of the blastocyst and is highly expressed in the placenta. In the adult, expression is restricted mostly to stratified epithelia of the skin and squamous epithelia of the upper gastrointestinal tract (Claas *et al*, 1996). At the mRNA level, a very similar expression profile of human (h) C4.4A was seen (Smith *et al*, 2001; Würfel *et al*, 2001). Recently, an antibody against hC4.4A and mouse (m) C4.4A was described (Hansen *et al*, 2004). With this antibody, expression of hC4.4A in placenta and skin was confirmed. In addition, mC4.4A expression was found to become upregulated in migrating keratinocytes during wound healing. In urothelium, too, C4.4A was described as an inducible wound response gene (Smith *et al*, 2001).

Although C4.4A expression is rather restricted in non-transformed tissues, C4.4A expression has been observed in several tumour entities. hC4.4A mRNA was detected in cancer cell lines of different origin including melanoma, breast, bladder and renal cell carcinoma (RCC) as well as in tumour tissues of malignant melanoma, breast cancer, lung carcinoma and lung tumour-derived metastases, and primary and metastatic transitional cell carcinoma of urothelial cell origin (Seiter *et al*, 2001; Smith *et al*, 2001; Würfel *et al*, 2001; Fletcher *et al*, 2003). Notably, and in line with the suggestion by Smith *et al* (2001) that C4.4A accounts for an inducible wound response gene in urothelial cells, we observed inducibility of the C4.4A gene (gene symbol: LYPD3) in malignant melanoma (Seiter *et al*, 2001).

To further pursue the question of C4.4A as a potential tumour marker, we generated hC4.4A-specific antibodies to characterize the molecule as expressed under physiological conditions and in tumour tissue. We provide evidence for differences in C4.4A glycosylation, oligomer status and associated proteins. We also show that C4.4A is released from tumour cells. Taking the restricted expression in non-transformed tissue and high-level expression in primary colorectal cancer and liver metastasis, C4.4A might well be suited as a diagnostic colorectal cancer marker.

## MATERIALS AND METHODS

### Tissues and tumour lines

Colorectal carcinoma, normal colorectal and colitis ulcerosa tissue, liver metastasis of colorectal cancer and normal liver were collected during surgery and snap-frozen in liquid nitrogen. The mean age of patients with colorectal cancer (72 male and 25 female) was 64 and ranged from 38 to 90 years. A collection of snap-frozen tissues from normal kidney and RCC as well as from normal pancreatic gland, chronic pancreatitis and pancreatic cancer has recently been described (Gesierich *et al*, 2005; Devitt *et al*, 2006). The analysis of human healthy skin has been performed with paraffin-embedded biopsy specimens, taken for routine histopathology. Informed consent for tissue collection was obtained from each patient and tissue collection was approved by the University Ethics Review Board. The rat cell lines BSp73ASML (ASML) (Zöller *et al*, 1978), Progressor (Reisser *et al*, 1993) and 804G (Izumi *et al*, 1981) were grown in RPMI medium supplemented with 10% FCS. The embryonic kidney cell line HEK-293 was grown in Iscove's medium supplemented with 10% FCS. The pancreatic adenocarcinoma lines BxPC3, Capan1, Capan2, Colo357, MiaPaca2, Panc1, Panc89 and 8.18 were maintained in RPMI 1640, supplemented with 10% FCS and 10 mM sodium pyruvate. The colorectal cancer lines Colo205, Colo320, Colo320DM, HT29, Lovo, SW480, SW707, SW948 and WIDR were maintained in RPMI 1640 and supplemented with 10% FCS. The origin of all these lines and the original depositors have recently been summarized (Gesierich *et al*, 2005). The prostate cancer cell lines LNCaP, PC-3 and DU145 were grown in RPMI 1640 and supplemented with 10% FCS. The breast cancer cell lines MCF-7, MELN, HCC, MDA-MB4361, BT4TD were grown in DMEM with 10% FCS. The HaCaT cell line (Boukamp *et al*, 1988) was grown in DMEM with 10% FCS. All lines grew adherent and, when confluent, were detached with trypsin for subculture. For the evaluation of activation-dependent expression of C4.4A, tumour lines were starved overnight in RPMI 1640 medium not containing foetal bovine serum. Thereafter they were cultured for 24 h in medium containing 10% AB0 serum that was or was not heat-inactivated for 30 min at 56°C (Seiter *et al*, 2001).

### Cloning of expression constructs and transient transfection of cells

A hC4.4A expression construct was generated by amplification of the hC4.4A coding sequence (Würfel *et al*, 2001) by PCR and cloning in the pCDNA3.1 vector (Invitrogen, Karlsruhe, Germany). HEK-293 cells were transiently transfected using the Polyfect transfection reagent (Qiagen, Hilden, Germany) as described by the manufacturer.

### Antibodies

The following monoclonal and polyclonal antibodies were used: C4.4 (mouse anti-rat C4.4A) (Matzku *et al*, 1989); CO-029 (anti-human CO-029) (Sela *et al*, 1989); HEA125 (anti-human EpCAM) (Momburg *et al*, 1987); anti-human uPAR (American Diagnostics, New York, NY, USA); rabbit anti-galectin-3 (Santa Cruz, Santa Cruz, CA, USA); biotin-conjugated anti-rabbit (Jackson ImmunoResearch, West Grove, PA, USA); and anti-mouse HRP (horseradish peroxidase), anti-rabbit HRP, streptavidin-HRP and anti-rabbit-phycoerythrin (PE) (all Dianova, Hamburg, Germany). Anti-hC4.4A polyclonal antibodies were raised in rabbits immunised with peptides QTPRQGVEHEASRDEEPRLTGGAAG and LTSRALDPAGNESAYPPNGV coupled to keyhole limpet hemocyanin (KLH). Antibodies were purified by passage of the antisera over a Sepharose 4B column that had been coupled to the peptides according to the manufacture's protocol (Amersham Biosciences Europe GmbH, Freiburg, Germany).

### Immunohistochemistry

Formalin-fixed, paraffin-embedded tissues were deparaffinised by two 5 min washes in xylene, then rehydrated through successive graded ethanol solutions and washed for 5 min in PBS. Antigen retrieval was achieved by immersing the slides in 0.01 M EDTA and boiling for 10 min in a water bath. Frozen sections were fixed 5 min in methanol/acetone (1 : 1). Endogenous hydrogen peroxidase activity was quenched by treating the slides in 3% hydrogen peroxidase/PBS for 10 min followed by two washes in PBS. The tissue was blocked in 10% BSA/PBS for 1 h. To avoid background staining due to the high content of mucus in colon and colorectal cancer tissue, slides were, in addition, pre-incubated for 30 min with 2% serum corresponding to the species of the secondary antibody before the addition of primary antibodies, which have been used at half the concentration applied for staining of non-colorectal tissue. Following three washes in PBS, the tissue sections were incubated with biotin-conjugated secondary antibody (Jackson ImmunoResearch) (5 *μ*g ml^−1^, 30 min) and alkaline phosphatase-conjugated avidin–biotin complex (Vector Laboratories, Grunberg, Germany) solutions (5 min). Tissue sections were counterstained with Mayer's haematoxylin. Staining was assessed semi-quantitatively by two independent observers according to the following score: no stained cells: −; weak staining or staining of less than 25% of cells: ±/0.5; moderate staining or staining of less than 50% of cells: +/1; moderate to strong staining of mostly >50% of cells: ++/2; strong staining of >75% of cells: +++/3. Tissues were considered as positive when both observers scored the tissue section as +/1. As far as the judgement of the two observers differed, new sections were stained and re-evaluated. These tissues were only considered as positive when both observers judged the repeated staining with +/1. As far as the judgement of both observers differed again, the tissue sample was not included in the final evaluation.

### FACS analysis

Flow cytometry followed routine procedures using 1−3 × 10^5^ tumour cells per sample. Trypsinized cells were allowed to recover for 2 h at 37°C in RPMI 1640 with 10% FCS. The primary antibody was used at a concentration of 5 *μ*g ml^−1^ and was replaced by normal rabbit IgG in the negative control. The secondary antibody was PE- or APC-labelled. Samples were analyzed using an FACS Calibur and the Cell Quest Program (Becton Dickinson, Heidelberg, Germany).

### Immunoprecipitation

Cells (1 × 10^7^) were surface-biotinylated with 1 mg ml^−1^ biotin-X-NHS WS (Calbiochem, San Diego, CA, USA) in HEPES puffer (25 mM HEPES, 150 mM NaCl, 5 mM MgCl_2_ (pH 7.4)) for 30 min at room temperature and lysed for 1 h at 4°C in 4 ml HEPES puffer with 1% Triton X-100 and a protease inhibitor cocktail (Roche Diagnostics GmbH, Mannheim, Germany). After centrifugation for 30 min at 20 000 **g**, lysates (1 ml) were precleared by incubation with 1/10 volume protein G-Sepharose for 2 h at 4°C. Pre-cleared lysates were incubated 3 h at 4°C with 1 *μ*g of antibody or control IgG. Immune complexes were washed four times with lysis buffer and precipitated proteins were analyzed by SDS–PAGE, followed by western blot (WB).

### Western blot

Proteins were resolved in 12% SDS–PAGE under non-reducing or reducing conditions and the proteins were transferred to PVDF membrane at 30 V overnight. After blocking (5% fat-free milk powder in PBS), membranes were incubated with streptavidin-HRP or with the indicated primary antibodies, followed by HRP-coupled secondary antibodies. Blots were developed with the enhanced chemiluminescence detection system (Amersham Biosciences Europe GmbH).

### Galectin-3 pull-down

Glutathione-*S*-transferase (GST)-galectin-3 was purified as described previously (Paret *et al*, 2005). For the GST pull-down, GST-galectin-3 fusion protein and GST, respectively, were immobilized on glutathione (GSH)-sepharose beads and allowed to interact for 2 h at 4°C with whole-cell lysate from the indicated cell lines. Following binding, GSH-sepharose beads were washed extensively with PBS containing 1% Triton X-100 and proteins were released by addition of 100 mM lactose. Released proteins were analysed by SDS–PAGE followed by WB. Where indicated, released proteins were deglycosylated before loading on SDS–PAGE gel.

### Detection of released C4.4A

Microvesicles were prepared by multi-step centrifugation. Cell culture supernatants were centrifuged two times at 800 **g**, followed by centrifugation at 1600 and 6000 **g**. Finally, microvesicles were precipitated by ultracentrifugation at 200 000 **g** and resuspended in PBS. Were indicated, microvesicles were subjected to deglycosylation before further analysis. Phase separation of C4.4A in Triton X-114 was performed as follows. After multi-step centrifugation (800, 1600 and 6000 **g**), cell culture supernatant was immunoprecipitated with C4.4 antibody. Immunoprecipitated C4.4A was eluted from the protein G-antibody complex using 100 mM glycine at pH 2. After neutralisation, the solution was adjusted to 2% Triton X-114 and incubated for 1 h at 4°C. Samples were cleared by centrifugation at 20 000 **g** for 10 min at 4°C. The supernatant was incubated for 5 min at 37°C. The detergent-rich and aqueous phases were separated by centrifugation for 5 min at 20 000 **g** at room temperature. Detergent-rich and aqueous phases were adjusted to identical volumes using PBS and analyzed for the presence of C4.4A by SDS–PAGE followed by WB.

### Trypsin digestion

Recombinant rC4.4A (rrC4.4A) carrying a myc tag at the C terminus (Paret *et al*, 2005) was digested for 2 h at 37°C with trypsin at the indicated concentrations.

### Deglycosylation

*In vivo* deglycosylation was performed by adding tunicamycin (SIGMA, Steinheim, Germany) at 5 *μ*g ml^−1^ to the cell culture medium overnight. For *in vitro* deglycosylation, cell lysates, immunoprecipitates, microvesicles or precipitates after pull-down were incubated for 16 h at 37°C with N-glycosidase F (20 U ml^−1^; ROCHE, Mannheim, Germany) and/or O-glycosidase (2.5 U 100 ml^−1^; ROCHE) and/or neuraminidase (0.05 U ml^−1^; SIGMA) in PBS containing 1% Triton X-100 before analysis by SDS–PAGE.

### Statistics

According to the specific question, association between quantitative and ordered variables was quantified by Spearman's rank correlation; the Jonckheere–Terpstra test for trend was used to investigate a trend in proportions; and the Wilcoxon rank sum test was used for two-group comparisons of quantitative variables. The signed rank test was used to compare paired quantitative observations. All tests were performed two-sided to the 0.05 level. Ninety-five percent confidence intervals were calculated for mean score differences. Sensitivity was defined as true positive (true positive plus false negative) and specificity as true negative (true negative plus false positive). The true positive rate is defined as the percentage of marker-positive tumour samples, the false positive rate is the percentage of marker-positive control samples, the true negative rate is the percentage of marker-negative control samples and the false negative rate is the percentage of marker-negative tumour samples. Sensitivities and specificities of different markers were compared by the *χ*^2^-test. All calculations were performed using SAS version 9.1 (SAS Institute Inc. Corp, NC, USA).

## RESULTS

### Characterisation of rabbit polyclonal antibodies against hC4. 4A peptides

**Figure 1:**
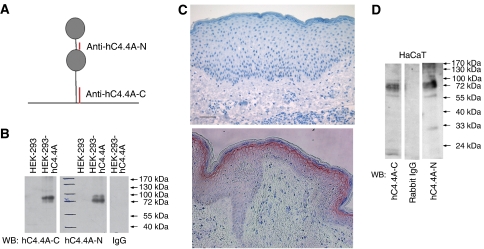
Production of antibodies against hC4.4A. (**A**) Schematic representation of the structure of hC4.4A. Circles represent the Ly-6 domains. Parts of the molecule recognised by the antibodies are in red. (**B**) HEK-293 cells were transfected with cDNA coding for hC4.4A (HEK-293-hC4.4A) or with vector alone (HEK-293). Lysates were analysed in WB with anti-hC4.4A-C (left) and anti-hC4.4A-N (middle). The right lane shows HEK-293-hC4.4A lysates blotted with rabbit IgG. (**C**) Immunohistochemistry of paraffin-embedded, normal healthy skin using rabbit IgG (negative control) and anti-hC4.4A-C at the concentration of 1.5 *μ*g ml^−1^. Scale bar=50 *μ*m. (**D**) Western blot of HaCaT cell lysate with anti-hC4.4A-C (left), anti-hC4.4A-N (right) and rabbit IgG (middle) (negative control).

Antibodies were generated by vaccination of rabbits with KLH-coupled hC4.4A peptides located at the C terminus (anti-hC4.4A-C) and N terminus (anti-hC4.4A-N) (Figure 1A). The hC4.4A peptides were coupled to sepharose, and C4.4A-specific antibodies were isolated from the antisera by passage over the peptide-coupled sepharose. The specificity of the antibodies was further verified by transfecting HEK-293 cells with hC4.4A cDNA. hC4.4A expression was analysed by WB. Only HEK-293 cells transfected with the hC4.4A cDNA showed a band of the expected size, whereas no reactivity was observed with lysates of HEK-293 cells transfected with the empty vector (Figure 1B). The anti-hC4.4A-C antibody stained paraffin-embedded specimens of healthy human skin, where expression of hC4.4A was restricted to the stratum granulosum. No staining was seen in the sections using a control IgG (Figure 1C). Both antibodies recognised the hC4.4A protein in WB in the immortalized keratinocyte line HaCaT (Figure 1D), which had been shown before by *in situ* hybridization to be C4.4A-positive (Würfel *et al*, 2001).

### Expression of hC4.4A in cancer lines

**Figure 2:**
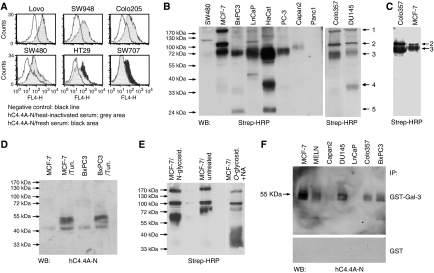
Expression of hC4.4A in cancer cell lines. (**A**) C4.4A expression was evaluated in colorectal cancer lines, which were cultured in heat-inactivated (grey area) or fresh ABO serum (black area). Overlays with the negative control (normal rabbit IgG/anti-rabbit APC) are presented. (**B**) Biotinylated lysates of the indicated cell lines were immunoprecipitated using anti-hC4.4A-C. Immunoprecipitates were boiled, separated by SDS–PAGE and blotted with streptavidin-HRP. Depending on the cell line, several bands were recovered (indicated by arrows), which represent different C4.4A isoforms and/or associated proteins (see Results). (**C**) Biotinylated lysates of Colo357 and MCF-7 cells were immunoprecipitated using anti-hC4.4A-C, and immunoprecipitates were boiled in the presence of 2-ME before SDS–PAGE and blotting with streptavidin-HRP. The high molecular weight band of about 200 kDa was not recovered after 2-ME treatment. (**D**) Where indicated, MCF-7 cells were treated with tunicamycin (Tun). Lysates were separated by SDS–PAGE and blotted with anti-hC4.4A-N. The antibody recognizes only lysates from Tun-treated cells. (**E**) Lysates of MCF-7 cells were biotinylated and immunoprecipitated with the anti-hC4.4A-C antibody. Where indicated, immunoprecipitates were treated with N-glycosidase F (N-glycosid.) or with O-glycosidase (O-glycosid.) plus neuraminidase (NA) before SDS–PAGE separation and WB analysis with streptavidin-HRP. O-glycosidase plus NA treatment resulted in a size reduction of C4.4A from 75 to 35 kDa–40 kDa. (**F**) Cell lysates of the indicated lines were incubated with GST-galectin-3 or GST. Bound proteins were eluted with 100 mM lactose. Eluted proteins were treated with N-glycosidase F before SDS–PAGE and blotting with anti-hC4.4A-N. C4.4A with a molecular weight of about 55 kDa was recovered in the precipitate of 5 out of 7 lines, but only after incubation with GST-galectin-3 and not after incubation with GST.

C4.4A expression on cancer cell lines was revealed by flow cytometry using the anti-hC4.4A-C antibody. Five breast, three prostate, five of eight pancreas and five of nine colorectal cancer lines were positive for hC4.4A (Table 1). Because C4.4A, similar to uPAR expression (Mazumdar *et al*, 2001), has been described to become upregulated or induced by, for example, heat-labile serum factors (Seiter *et al*, 2001), expression of C4.4A on colorectal cancer lines was re-evaluated after cells had been cultured for 24 h in the presence of fresh AB0 serum. Expression in the C4.4A-positive lines mostly remained unaltered, but C4.4A expression was induced by heat-labile serum factors in colorectal cancer lines that in the resting state did not or very weakly express C4.4A (Table 1 and Figure 2A). hC4.4A expression was confirmed by immunoprecipitation of biotinylated lysates (Figure 2B, left), which revealed significant differences in the molecular weight of hC4.4A. The band indicated with arrow 3 corresponds in the molecular weight to the monomeric form of hC4.4A. Changes in the molecular weight of this band between different cell lines are probably due to differences in the glycosylation state. After *in vivo* deglycosylation with tunicamycin, an inhibitor of N-glycosylation, a band of about 50 kDa is detectable by WB analysis in MCF-7 and BxPC3 cell lysates using the anti-hC4.4A-N antibody. A size reduction was also observed after *in vitro* deglycosylation with N-glycosidase F of immunoprecipitated hC4.4A from the MCF-7 cell line (Figure 2E). The expected size of the protein as calculated from the amino-acid composition is 32 kDa. Thus, O-glycosylations likely account for the difference to the expected size. In the presence of O-glycosidase alone, protein size was unchanged (data not shown), indicating that O-glycosylations carry sialic acid modifications. Indeed, in the presence of neuraminidase and O-glycosidase, the protein size was reduced to a broad band of 35–40 kDa (Figure 2E).

Notably, the fully glycosylated C4.4A isoform as expressed in MCF-7 and BxPC3 cells is not recognized by both antibodies in WB, as demonstrated for hC4.4A-N (Figure 2D), but both antibodies reacted well with hC4.4A on the HaCaT cell line (Figure 1D). Because the hC4.4A-N antibody did react with the tumour line lysates after N-deglycosylation, it is likely that C4.4A glycosylation differs in HaCaT cells and the tested tumour lines, where in the latter the binding sites may be masked by glycosylation. Flow cytometry analysis of C4.4A expression in colorectal cancer lines was also repeated after O- and N-deglycosylation. In most lines, staining intensity was at least slightly increased after O- or N-deglycosylation. Staining intensity was most strongly increased after N-deglycosylation of Colo205 and after O-deglycosylation of Lovo (data not shown).

We previously described that rat C4.4A interacts with galectin-3 (Paret *et al*, 2005). Because binding depends on the carbohydrate residues of C4.4A, we wondered if all glycosylation forms of C4.4A bind galectin-3. In fact, only 5 of 7 human cancer cell lines express a C4.4A isoform, which allows galectin-3 binding (Figure 2F).

Immunoprecipitation of biotinylated human cancer cells with anti-hC4.4A-N uncovered additional features that differed between the individual lines. The higher molecular weight band, seen with MCF-7, Colo357 and, albeit weakly, with DU145 and LNCaP cell lysates (Figure 2A, arrow 1) possibly is a C4.4A oligomer, because it disappears after reduction with 2-mercaptoethanol (2-ME) as demonstrated for MCF-7 and Colo357 (Figure 2B). Moreover, in the presence of 2-ME, the monomeric form of Colo357 and MCF-7 runs at a slightly higher molecular weight. This could be due to the reduction of the disulphide bridges contained in the two Ly-6 domains and the loss of the three-dimensional folding. Interestingly, the putative C4.4A oligomer was detected only in cancer cell lines, which according to the flow cytometry analysis displayed high hC4.4A expression. A band of about 100 kDa (Figure 2A and B, arrow 2) could represent a highly glycosylated form of hC4.4A or an interacting protein. The latter is more likely, because the band is still visible after deglycosylation (Figure 2D). Two additional co-immunoprecipitating proteins of about 38 and 25 kDa were detected in the precipitate of the HaCaT cell line and albeit weakly in DU145 (35 kDa) and BxPC3 (25 kDa) cell lysates (arrow 4 and 5). Because cells were labelled with membrane impermeable biotin, these two interacting proteins are likely membrane-associated. Identification of these associating proteins that are absent or very weakly expressed in tumour lines is in progress.

Taken together, human tumour cell lines frequently express highly glycosylated C4.4A, which obviously can prevent binding of the antibodies generated by peptide vaccination and can also interfere with the association of galectin-3 with C4.4A. High C4.4A expression can also lead to oligomer formation. The consequences of the glycosylation-prohibited associations as well as of oligomer formation on the function of C4.4A remain to be explored.

### Expression of C4.4A and galectin-3 in colorectal, pancreatic and RCC

C4.4A expression was evaluated in frozen specimen of colorectal cancer, liver metastasis of colorectal cancer, pancreatic adenocarcinoma and RCC by immunohistochemistry. Normal colon and normal liver do not (98 and 97%, respectively) or very weakly (2 and 3%, respectively) express hC4.4A. Yet, >80% of colon cancer and >70% of liver metastasis show distinct to strong staining. Importantly, C4.4A expression was not induced in inflamed (colitis ulcerosa) tissue of the colon. While kidney and pancreatic gland tissue also did not or weakly express C4.4A, C4.4A expression was observed in 47% of RCC and 53% of pancreatic adenocarcinoma. However, C4.4A expression was also seen in 40% of chronic pancreatitis tissue (Table 2A). To validate the reliability of C4.4A as a tumour marker, particularly for colorectal cancer, expression of C4.4A was compared with galectin-3, EpCAM and CO-029 expression, that all three have been described to be upregulated in colorectal cancer (Sela *et al*, 1989; Armstrong and Eck, 2003; Nagy *et al*, 2003). In primary colorectal cancer, the sensitivity for C4.4A was 0.85 (95% CI: 0.73–0.84) and the specificity 1.00 (95% CI: 0.94–1.00). The sensitivity for galectin-3 was 0.79 (95% CI: 0.70–0.87) and the specificity 0.97 (95% CI: 0.91–0.99). The sensitivity for EpCAM was 0.94 (95% CI: 0.87–0.98) and the specificity 0.80 (95% CI: 0.71–0.87). The sensitivity for CO-029 was 0.98 (95% CI: 0.92–1.00) and the specificity 0.94 (95% CI: 0.86–0.98) (Kuhn *et al*, 2007). Thus, sensitivity of EpCAM was slightly (*P*=0.08) and of CO-029 was significantly higher (*P*=0.004) than for C4.4A. However, the specificity of EpCAM was significantly reduced compared with C4.4A (*P*=0.002). The corresponding values for liver metastasis of colorectal cancer are C4.4A: 0.79 sensitivity (95% CI: 0.63–0.90), 1.00 specificity (95% CI: 90–100%); galectin-3: 0.83 sensitivity (95% CI: 0.70–0.91), 1.00 specificity (95% CI: 0.94–1.00); EpCAM: 0.93 sensitivity (95% CI: 0.83–0.98), 0.91 specificity (95% CI: 0.81–0.97); and CO-029: 0.94 sensitivity (95% CI: 0.85–0.99), 0.94 specificity (95% CI: 0.85–0.99) (Kuhn *et al*, 2007). Accordingly, in liver metastasis of colorectal cancer, the sensitivity of EpCAM and CO-029 also was significantly higher than for C4.4A (*P*=0.04 and 0.02, respectively). The specificity of EpCAM was slightly reduced compared with C4.4A.

There have been too few colitis ulcerosa tissues to warrant statistical analysis. It should, however, be mentioned that, distinct to C4.4A (Table 2A), galectin-3 expression was variable in colitis ulcerosa. Three out of six tissue samples showed no staining, two samples were weakly stained and one sample was distinctly stained (data not shown).

Staining of ductal cells of the pancreatic gland with anti-CO-029 had been observed in 50% and of pancreatic cancer tissue in 100% (Gesierich *et al*, 2005). EpCAM expression was not only seen in 87% of pancreatic adenocarcinoma, but also in 87% of the pancreatic gland. However, pancreatic gland tissue was not stained by anti-uPAR, a marker discriminating between chronic pancreatitis and pancreatic adenocarcinoma (Chen *et al*, 2007), whereas 67% of pancreatic adenocarcinoma were stained. The data correspond to sensitivity values of C4.4A: 0.53, galectin-3: 0.80, EpCAM: 0.87, CO-029: 1.00 and uPAR: 0.67. Accordingly, the sensitivity of galectin-3 (*P*=0.03), EpCAM (*P*=0.005) and CO-029 (*P*<0.0001) was significantly higher than the sensitivity of C4.4A. However, the specificity of galectin-3 (0.75) was equal (*P*=1), and the specificity of EpCAM (0.13) (*P*=0.0004) and CO-029 (0.50) (*P*=0.02) was significantly lower than that of C4.4A (1.00). Urokinase receptor also showed a sensitivity of 1.00.

Also, 38% of RCC sections, which differentially express uPA and uPAR (Bhuvarahamurthy *et al*, 2005), were stained by anti-uPAR; kidney sections were not stained. Thus, uPAR and C4.4A showed a specificity value of 1.00. The sensitivity values of 0.57 for C4.4A and 0.38 for uPAR differed significantly (*P*=0.009).

It should be kept in mind that we used suboptimal concentrations of all antibodies for the staining of colonic mucosa and colorectal cancer tissue to avoid unspecific staining. As we observed staining of colorectal cancer tissue with all four antibodies in 79–98%, it implies that irrespective of the possible underestimate particularly of EpCAM and galectin-3 expression in normal tissue, expression of these four molecules is significantly upregulated in colorectal cancer such that expression in primary colorectal cancer and that in liver metastasis differ at a highly significant level from expression in the corresponding normal tissue. For C4.4A expression, this also accounts for the comparison to inflamed tissue (Table 2A and B).

**Figure 3:**
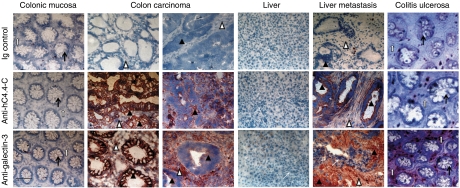
Expression of hC4.4A and galectin-3 in colorectal cancer and liver metastasis. Immunohistochemistry of the indicated tissues was performed with anti-hC4.4A-C, anti-galectin-3 or rabbit IgG (negative control) at the concentration of 5 *μ*g ml^−1^ (colon) and 10 *μ*g ml^−1^ (liver). Representative examples are shown. Open arrow, mucosa epithelium; white-filled arrow, submucosa; black arrowhead, tumour cells; white arrowhead, tumour stroma; thin white arrow, hepatocytes. Anti-hC4.4A-C stains tumour cell membranes, and anti-galectin stains tumour cell membranes and tumour stroma. Scale bar=50 *μ*m.

Finally, we want to point out, as exemplified in Figure 3, that C4.4A expression was restricted to the tumour cell membrane. Galectin-3 was predominantly expressed in the tumour cells, but was also detected in the tumour stroma of some tissue samples. In colitis ulcerosa, galectin-3 expression was restricted to stromal cells (Figure 3).

Considering C4.4A expression in pancreatic cancer and RCC, the sensitivity values of 0.53 and 0.57 rather excluded C4.4A in these tumour entities as a diagnostic marker. Therefore, additional statistical analyses on a potential correlation between C4.4A and galectin-3 expression and clinical parameters of grading, staging and disease-free survival were performed only for colorectal cancer and liver metastases derived thereof. As could have been expected by the high frequency and the high level of C4.4A and galectin-3 expression in colorectal cancer, expression of both molecules did not become upregulated at a statistically significant level in dependence on tumour staging, the involvement of lymph nodes and distant organs. C4.4A and galectin-3 expression also did not significantly vary depending on tumour grading (Table 2C). Finally, C4.4A and galectin-3 expression did not correlate at a statistically significant level with the disease-free survival (Table 2D).

### C4.4A is released in the supernatant of cancer cells lines

**Figure 4:**
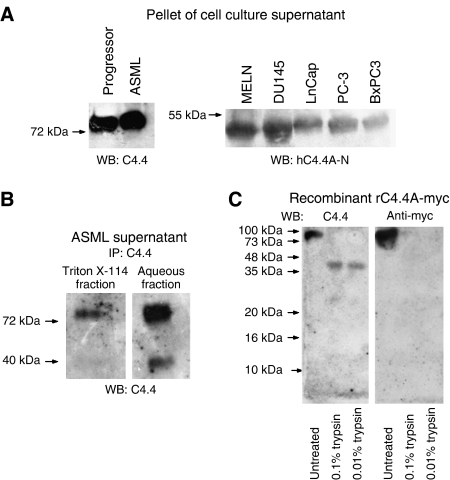
Release of rat and human C4.4A. (**A**) Supernatant of the indicated cell lines was subjected to ultracentrifugation. The pellets were separated by SDS–PAGE and blotted with C4.4 (left) or anti-hC4.4A-N (right). For analysis with hC4.4A-N, pellets were treated with N-glycosidase F before SDS–PAGE. C4.4A was recovered in the pellet (vesicles containing uncleaved C4.4A). (**B**) Supernatant of ASML cells was subjected to IP with C4.4. Bound proteins were eluted and extracted with Triton X-114. The Triton X-114 fraction (left) and the aqueous fraction (right) were separated by SDS–PAGE and blotted with C4.4. The Triton X-114 fraction contained a considerable amount of C4.4A (GPI anchor-containing C4.4A). Two bands of about 80–90 and 40 kDa were recovered from the aqueous fraction, the lower molecular weight band indicating a proteolytic cleavage product of C4.4A. (**C**) Rat recombinant C4.4A with a C-terminal myc tag was treated for 2 h at 37°C with 0.01 or 0.1% trypsin. After SDS–PAGE of the undigested and trypsin-digested rrC4.4A protein, blots were incubated with C4.4 (left) and anti-myc (right). The undigested 90 kDa recombinant protein was detected with both C4.4 and anti-myc. Only C4.4 recognized a fragment of about 40 kDa.

The C4.4A-related uPAR molecule mostly is shed without a GPI anchor (Ploug *et al*, 1992; Wilhelm *et al*, 1999), but uPAR fragments have also been described (Sidenius *et al*, 2000; Montuori *et al*, 2005; Beaufort *et al*, 2007); uPAR with an intact GPI anchor is also detected in shed membrane vesicles (Wilhelm *et al*, 1999). To analyse whether C4.4A is present in shedded membrane vesicles, supernatant of two rat and five human tumour lines was subjected to multi-step centrifugation for elimination of cell debris followed by ultracentrifugation to collect membrane vesicles. The rat tumour lines were included because the rat, different from the human C4.4A-specific antibodies, has been shown to recognize C4.4A indendently of gylcosylation and, in addition, is well suited for immunoprecipitation (Matzku *et al*, 1989; Rösel *et al*, 1998). C4.4A was found in the vesicles containing pellet of all lines (Figure 4A). To see whether the GPI anchor is cleaved during C4.4A release, ASML supernatant was concentrated and immunoprecipitated after cell debris elimination. The immunoprecipitate was subjected to Triton X-114 extraction. Triton X-114-containing solutions are homogenous at 4°C, but separate into detergent-rich and aqueous phases at 37°C. Glycosylphosphatidylinositol anchor containing C4.4A, probably vesicle-associated, partitioned into the detergent-rich phase (Figure 4B, left). However, the major part of released rC4.4A partitioned into the aqueous phase (Figure 4B, right), indicating GPI anchor hydrolysis and removal of the hydrophobic portion of the anchor. In the water-soluble fraction, an additional rC4.4A fragment of about 40 kDa was detected (Figure 4B), which could represent a proteolytic cleavage product of C4.4A. To support this hypothesis, rrC4.4A carrying a myc tag at the C terminus was digested with trypsin. Thereafter, the proteins were separated by SDS–PAGE and blotted with C4.4 and anti-myc. Trypsin-treated rrC4.4A revealed a 40 kDa protein, when blotted with C4.4. Only the undigested rrC4.4A was detected with the anti-myc antibody, indicating that the fragment represents the N-terminal part of C4.4A (Figure 4C). In fact, a protease-sensitive region between domains 2 and 3 of C4.4A has recently been described (Hansen *et al*, 2004). Thus, similar to uPAR, cancer cells release C4.4A with its GPI anchor in shed vesicles, as soluble, GPI anchor cleaved molecule and as fragments thereof.

## DISCUSSION

The GPI-anchored C4.4A glycoprotein was first identified in a highly metastasizing rat pancreatic adenocarcinoma line (Matzku *et al*, 1989). The possible importance of the protein as tumour marker was indicated by the restricted expression in normal tissues (Rösel *et al*, 1998; Smith *et al*, 2001; Würfel *et al*, 2001) and upregulation of C4.4A mRNA in different tumours (Seiter *et al*, 2001; Smith *et al*, 2001; Fletcher *et al*, 2003). We here report hC4.4A protein expression in several tumour lines and tumour tissues and provide first evidence suggestive for C4.4A as a possible serum marker for cancer diagnosis.

### Glycosylation, oligomer formation and interaction partners of C4.4A in human tumour lines

Two polyclonal antibodies were raised against peptides localised at the C terminus and between domains 1 and 2, respectively, of human C4.4A. The antibody against the C terminus stained the stratum granulosum of healthy human skin. A recently described polyclonal hC4.4A-specific antibody that cross-reacts with mC4.4A stained the suprabasal layer of the epidermis (Hansen *et al*, 2004). In the rat, C4.4A is strongly expressed by basal keratinocytes, which are in contact with the extracellular matrix, and staining decreases in suprabasal keratinocytes. These different staining profiles might be due to species differences. It is also possible that differences are due to the epitopes recognized by the different antibodies. Only for the generation of the monoclonal anti-rC4.4A antibody, mice were vaccinated with cell membranes (Matzku *et al*, 1989). This antibody obviously recognizes an epitope that does not become hidden by glycosylation (Rösel *et al*, 1998). Instead, a recombinant protein vaccination-derived antibody (Hansen *et al*, 2004) and our peptide vaccination-derived antibodies did not stain basal keratinocytes. However, these antibodies stained HaCaT cells. Because glycosylation of C4.4A shows great variability and glycosylation of keratinocytes depends on the differentiation status (Mehul *et al*, 2003), it becomes likely that the restricted epidermal staining patterns by distinct anti-C4.4A antibodies are a consequence of C4.4A glycosylation that may hide the respective epitopes.

In fact, C4.4A carries N- and O-glycosylation sites, where the O-glycosylations are modified by sialic acid. Interestingly, while hC4.4A was detected by WB in HaCaT cell lysate, both antibodies did not react in WB with hC4.4A in cancer cell lines, but the hC4.4A-N antibody did so after deglycosylation. The finding suggests that C4.4A glycosylation differs between tumour cell lines and the immortalized keratinocyte line HaCaT, such that binding sites for anti-hC4.4A-N and probably anti-hC4.4A-C are frequently masked by sugar chains in tumour lines. Indeed, the peptide recognised by the anti-hC4.4A-N antibody has a potential N-glycosylation site at AA position 129 and the peptide recognised by the anti-hC4.4A-C antibody is near a potential O-glycosylation site (Hansen *et al*, 2004). We previously described that C4.4A interacts with galectin-3 (Paret *et al*, 2005), which is associated with colorectal cancer progression (Schoeppner *et al*, 1995; Nakamura *et al*, 1999; Endo *et al*, 2005). The interaction of galectin-3 with its cellular ligands depends on the glycosylation status of the interaction partner (Barondes *et al*, 1994). In fact, some of the glycosylated forms of hC4.4A as expressed on cancer cells did not bind galectin-3. Whether, indeed, differences in the C4.4A glycosylation state account for the distorted interaction with galectin-3 remain to be elucidated. However, altered glycosylation in cancer can play an important role in tumour cell adhesion and migration (Orntoft and Vestergaard, 1999), as well as in extravasation via interaction with galectin-3 on endothelial cells (Lehr and Pienta, 1998; Glinsky *et al*, 2000; Krishnan *et al*, 2005). Two additional membrane proteins that associate with C4.4A in HaCaT cells, but very rarely in tumour cells as well as a high molecular weight protein, mainly detected in MCF-7 cells, remain to be identified.

C4.4A likely can form oligomers, which are not detected after 2-ME treatment and are reduced in size after deglycosylation. Because oligomers were only detected in cells, which according to flow cytometry analysis expressed C4.4A at a high level, the monomer/oligomer status may depend on the expression level. Furthermore, oligomers disappeared in the presence of *β*-mercaptoethanol. Thus, they are possibly formed via disulfide bridges. Alternatively, the native conformation of the molecule is required for oligomerisation. Expression level and oligomerisation of uPAR determine whether uPA elicits an adhesive or migratory response (Chiaradonna *et al*, 1999), high level uPAR expression contributes to tumorigenicity and invasiveness (Aguirre Ghiso *et al*, 1999), and oligomerisation of uPAR is required for vitronectin binding (Wei *et al*, 1994; Sidenius *et al*, 2002). Whether the expression level of C4.4A and oligomerisation also has functional consequences remains to be explored.

### C4.4A as a candidate marker for the diagnosis of colorectal cancer

C4.4A is expressed at distinct to high levels in primary colorectal cancer as well as in liver metastasis in more than 80% of the patients with negligible expression in normal colonic mucosa and normal liver. Importantly, C4.4A expression was also largely absent in inflamed colonic mucosa and liver. C4.4A expression was compared with the expression of EpCAM, galectin-3 and CO-029, which have been described as reliable markers in colorectal cancer (Sela *et al*, 1989; Armstrong and Eck, 2003; Nagy *et al*, 2003). With respect to sensitivity, EpCAM (liver metastases, only) and CO-029 were superior to C4.4A. However, with respect to specificity, C4.4A proved to be superior to EpCAM and CO-029.

Notably, too, frequency and intensity of C4.4A expression apparently did not vary at a statistically significant level in dependence on tumour grading and staging, nor between primary tumours and metastasis. Therefore, one could argue that C4.4A expression is induced early during tumorigenesis and remains stable. However, our data do not allow to exclude upregulation during tumour progression. First, immunohistochemistry is a semi-quantitative method; second, we experienced that very high C4.4A expression can be accompanied by oligomerisation, which may prohibit recognition by the available antibodies; third, the antibodies also do not bind to highly glycosylated C4.4A. The fact that we observed at the RNA level a clear upregulation of C4.4A expression at the tumour boundary and in metastatic tissue of malignant melanoma (Seiter *et al*, 2001) rather argues for C4.4A expression levels to be regulated also in tumour tissue, which possibly becomes hidden by modifications of the molecule that prevent recognition by the available antibodies. Nonetheless, the high frequency of C4.4A expression in colorectal cancer and liver metastasis makes the molecule a candidate marker in the diagnosis of colorectal cancer.

Despite the high frequency of C4.4A expression in primary tumours and liver metastases, C4.4A expression was noted only in 5 of 9 colorectal cancer lines. This feature is in line with the notion that C4.4A expression is strictly regulated (Seiter *et al*, 2001; Smith *et al*, 2001; Hansen *et al*, 2004), requires a C/EBP*β*/JunD or c-Jun complex for induction of transcription (Fries *et al*, 2007) and can also be induced in tumour lines by not yet defined heat-labile serum factors (Würfel *et al*, 2001). These stimuli will be present *in vivo*, but be missed in culture. In fact, *de novo* C4.4A expression was seen in colorectal cancer lines after starving and culturing in fresh serum.

We also evaluated C4.4A expression in pancreatic cancer tissue, which had been tested before for CO-029 expression (Gesierich *et al*, 2005). C4.4A expression was only seen in 53% of pancreatic cancer tissue. Though it was absent in normal pancreatic tissue, C4.4A was also expressed in 40% of chronic pancreatitis. Thus, for pancreatic adenocarcinoma, uPAR expression (Chen *et al*, 2007) may be a more reliable parameter than C4.4A expression. In RCC, the vast majority of which were clear cell carcinoma (Devitt *et al*, 2006), both C4.4A and uPAR (Bhuvarahamurthy *et al*, 2005) expressions show low sensitivity. Still, C4.4A proved to be the more sensitive marker.

Finally, the finding that C4.4A is released by cancer cells may well become important for its potential use in colorectal cancer diagnosis. Different forms of released C4.4A were detected. Part of released C4.4A contains a GPI anchor and is likely vesicle-associated. Vesicle shedding can occur in different normal cells (Dainiak, 1991), but is strongly accelerated in cancer cells (Taylor and Black, 1986). Similar to uPAR (Ploug *et al*, 1992; Wilhelm *et al*, 1999), the majority of released C4.4A has no GPI anchor, possibly due to the activity of GPI-specific phospholipase D (Wilhelm *et al*, 1999). The supernatant of the ASML tumour line also contained a C4.4A fragment, which corresponds in size to a cleavage product generated by digestion of rrC4.4A. The size of the fragment is compatible with C4.4A cleavage at a protease-sensitive region between the second and the third domain (Hansen *et al*, 2004). Interestingly, soluble uPAR and uPAR fragments are present in the blood of patients with different types of solid tumours, for example non-small cell lung cancer, breast, colorectal, prostate and ovarian cancer (Pappot *et al*, 1997; Stephens *et al*, 1997, 1999; Sier *et al*, 1998; Miyake *et al*, 1999), and are used as prognostic marker. Moreover, vesicles shed *in vivo* by breast carcinoma are similar to those shed *in vitro* by breast cancer cells and probably contribute to tumour markers present in the circulation of cancer patients (Dolo *et al*, 1995). Thus, it will be interesting to analyse the presence of hC4.4A in the serum and ascitic fluid of cancer patients and whether high enough amounts are present to allow for detection by a sensitive ELISA.

Besides C4.4A expression, we were particularly interested in galectin-3 expression, which we had described to be an interaction partner for C4.4A (Paret *et al*, 2005). Galectin-3 is also expressed in over 70% of colorectal primary tumours and liver metastasis. Distinct to C4.4A, which is expressed exclusively by the epithelial tumour cells, galectin-3 can also be expressed by stromal cells and expression becomes upregulated in colitis ulcerosa tissue. Yet, the differences between expression levels in inflamed and malignant colorectal tissue remained significant. This is in line with several reports (Byrd and Bresalier, 2004) on high-level expression of galectin-3 in colorectal cancer. We did, however, not observe significant differences in the intensity of expression between low- and high-grade tumours and not in dependence on the disease-free survival, although a prolonged disease-free survival was accompanied by a slight, statistically non-significant reduction in the mean intensity of expression (1.43) as compared to metastasis developing concomitantly with the primary tumour (mean intensity: 1.73). Other studies revealed either similar results or reported upregulation of galectin-3 expression during tumour progression (Andre *et al*, 1999; Nakamura *et al*, 1999; Legendre *et al*, 2003, Nagy *et al*, 2003; Endo *et al*, 2005). Particularly one of the latter studies performed a highly sophisticated quantitation of galectin-3 expression (Nagy *et al*, 2003). Taking into account that we observed a slightly lower galectin-3 expression in metastasis appearing after a prolonged disease-free survival, we would argue that probably galectin-3 expression becomes upregulated during tumour progression, but to a minor degree. Irrespective of this open question, and in line with several reports, galectin-3 still fulfils the criteria of a colorectal tumour marker with a sensitivity of 0.79 and 0.83 and a specificity of 0.97 and 1.00 in primary and metastatic tissue.

Our results suggest C4.4A as a candidate diagnostic marker for colorectal cancer. The possibility of C4.4A as tumour marker in body fluids remains to be explored. The advantage of C4.4A in colorectal cancer diagnosis relies not only on the high frequency and stability of expression, but particularly on the *de novo* expression of this molecule in colorectal cancer tissues or the strongly upregulated expression as for example in transitional cell cancer (Smith *et al*, 2001). In view of oligomerization and highly variable glycosylation of C4.4A, establishing a monoclonal antibody by vaccination with native, glycosylated C4.4A is desirable and may be mandatory for low-level detection of C4.4A in body fluids.

## Figures and Tables

**Table 1:** hC4.4A expression in human cancer cell lines (flow cytometry)

**C4.4A expression**								
**Pancreatic cancer**	**Intensity^a^**	**Colorectal cancer**	**Intensity^a^ unstimulated**	**Intensity^a^ stimulated^b^**	**Breast cancer**	**Intensity^a^**	**Prostate cancer**	**Intensity^a^**
Colo357	+++	SW480	+	+	MCF-7	+++	LNCaP	+++
Capan2	++	Colo205	+	+	MELN	+++	Du145	+++
Panc89	++	HT29	−	+	HCC1937	++	PC3	+
BxPC3	+	WIDR	−	+	BT47D	++		
MiaPaca2	+	Colo320	−	+	MDA-MB436	+		
Panc1	−	Colo320DM	+	+				
8.18	−	SW707	−	+				
Capan1	−	SW948	++	++				
		Lovo	++	++				

a Flow cytometry data were analysed according to the increase in the mean fluorescence intensity as compared to the negative control (normal rabbit IgG plus anti-rabbit IgG-PE): –(negative), intensity 1.0- to 1.5-fold; + (distinct), intensity 1.5- to 4-fold; ++ (strong), intensity 4- to 10-fold; +++ (very strong), intensity >10-fold.

b Cells were starved overnight and thereafter incubated for 24 h in RPMI 1640 containing 10% fresh AB0 serum.

**Table 2:** Expression of C4.4A in colorectal, pancreatic and renal cell carcinoma

*(A) Comparison between tumour and non-transformed tissue*							
		**% stained samples^a^**					
**Tissue**	**No. of samples**	**Negative (−)**	**Weak (±)**	**Distinct (+) C4.4A**	**Strong (++)**	**Very strong (+++)**	***P*-value^§^**
Colonic mucosa	61	98.4	1.6	0.0	0.0	0.0	
Colitis ulcerosa	6	100.0		0.0	0.0	0.0	
Colorectal carcinoma	55	7.3	7.3	23.6	32.7	29.1	<0.0001
Liver	35	97.1	2.9	0.0	0.0	0.0	
Liver metastasis	38	13.2	7.9	31.6	31.6	15.8	<0.0001
Pancreatic gland	8	62.5	37.5	0	0	0	
Chronic pancreatitis	10	40.0	20.0	10.0	20.0	10.0	
Pancreatic carcinoma	30	26.7	20.0	40.0	10.0	3.3	0.01
Kidney	10	70.0	30.0	0.0	0.0	0.0	
Renal cell carcinoma	61	21.3	21.3	31.1	16.4	9.8	NS

*(B) Comparison between different tumour markers*							
	**Marker**						
**Parameter**	**C4.4A**	**Galectin-3^†^**	**EpCAM^b^^,†^**	**CO-029^b^^,†^**	**UPAR^†^**		
*Colorectal cancer*							
No cancer tissue	55	100	100	93			
No control tissue	61	100	100	93			
Sensitivity^c^	0.85	0.79 (NS)	0.94 (NS)	0.98 (0.004)			
Specificity^c^	1.00	0.97 (NS)	0.80 (0.0002)	0.94 (0.045)			
							
*Liver metastasis*							
No cancer tissue	38	57	57	54			
No control tissue	35	57	57	54			
Sensitivity	0.79	0.83 (NS)	0.93 (0.04)	0.94 (0.02)			
Specificity	1.00	1.00 (NS)	0.91 (NS)	0.94 (NS)			
							
*Pancreatic cancer*							
No cancer tissue	30	30	30	30	30		
No control tissue	8	8	8	8	8		
Sensitivity	0.53	0.80 (0.03)	0.87 (0.005)	1.00 (0.00002)	0.67 (NS)		
Specificity	1.00	0.75 (NS)	0.13 (0.0004)	0.50 (0.02)	1.00 (NS)		
							
*Renal cell carcinoma*							
No cancer tissue	61				61		
No control tissue	10				10		
Sensitivity	0.57				0.38 (0.009)		
Specificity	1.00				1.00 (NS)		

*(C) Correlation to colorectal tumour grading and staging*							
**Primary tumour**	**No. of samples**	**C4.4A % stained (mean staining intensity)^d^**	***P*-value^+^**	**No. of samples**	**Galectin-3 % stained (mean staining intensity)^d^**	***P*-value^+^**	
*Primary tumour staging*							
T0–1	4	100 (2.00)	NS	6	100 (1.50)	NS	
T2	11	100 (2.50)		22	100 (1.75)		
T3	25	84.0 (1.68)		49	91.8 (1.73)		
T4	15	100.0 (1.68)		20	95.0 (1.74)		
							
*Lymph node staging*							
N0	35	94.3 (1.51)	NS	61	96.7 (1.66)	NS	
N1	12	91.7 (1.90)		23	100.0 (1.77)		
N2	8	87.5 (1.42)		13	76.9 (1.55)		
							
*Metastasis staging*							
M0/mix	43	93.0 (1.62)	NS	74	95.6 (1.69)	NS	
M1	12	91.7 (1.85)		23	91.3 (1.81)		
Primary tumour grading							
G0/G1	2	100.0 (2.20)	NS	5	100.0 (1.83)	NS	
G2	44	90.9 (1.86)		72	94.4 (1.72)		
G3/G4	9	88.9 (1.83)		20	95.0 (1.74)		

*(D) Correlation to the disease-free survival in colorectal cancer patients*							
**Disease-free survival (months)**	**No. of samples**	**C4.4A % stained (mean intensity of staining)^d^**	***P*-value^§§^**	**No. of samples**	**Galectin-3 % stained (mean intensity of staining)^d^**	***P*-value^§§^**	
*Primary tumour*							
0	13	84.6 (1.86)	NS	26	92.3 (1.81)	NS	
Undefined	42	92.9 (1.61)		71	98.8 (1.70)		
							
*Liver metastasis*							
0	11	100 (1.44)	NS	20	90.0 (1.73)	NS	
1–24	17	94.1 (1.44)		22	90.9 (1.43)		
>24	9	88.9 (1.55)		15	93.3 (1.43)		

NS=not significant.

a Mean intensity of staining was estimated as indicated in Materials and Methods; samples classified as − or ± were considered negative, samples classified as +, ++ and +++ were considered as positive.

b Part of these analyses has already been described (Gesierich *et al*, 2005; Kuhn *et al*, 2007).

c Sample with a score of − and ± were considered as negative; samples with a score of +, ++ and +++ were considered as positive; sensitivity=true positive: (true positive + false negative); specificity=true negative: (true negative + false positive).

d Mean intensity of staining was estimated as indicated in material and methods, e.g. a score of ± was taken as 0.5 and a score of +++ as 3.

§ Signed rank test; for pancreatic adenocarcinoma, Wilcoxon rank sum test.

† *P*-value=*χ*^2^-test.

+ The correlation between marker expression and tumour staging, lymph node staging, metastasis staging and tumour grading was calculated by the Jonckheere–Terpstra test for trend. No significant differences were observed.

^§§^Expression of C4.4A and galectin-3 did not differ significantly (Wilcoxon rank sum test) in primary tumours of patients who had developed liver metastasis concomitantly with the primary tumour of those who had not. In liver metastasis C4.4A and galectin-3 expression did not correlate with the disease-free survival.
